# Supplementary material for: Non-invasive imaging techniques for diagnosis of pelvic deep endometriosis and endometriosis classification systems: an International Consensus Statement
Source: Facts Views Vis Obgyn. 2024 Jun 28;16(2):127–44. doi: 10.52054/FVVO.16.2.012 (PMC11366111; doi:10.52054/FVVO.16.2.012)
Supplement: Figure SI — Revised American Society for Reproductive Medicine (rASRM) classification of endometriosis. Reprinted from the Revised American Society for Reproductive Medicine classification of endometriosis: 1996. Fertil Steril. 1997;67:817–21. Copyright© 1997 American Society for Reproductive Medicine, with permission from Elsevier. All rights reserved. [file FVVinObGyn-16-127-gs001.pdf]

AMERICAN SOCIETY FOR REPRODUCTIVE MEDICINE
REVISED CLASSIFICATION OF ENDOMETRIOSIS

Patient's Name ______________________ Date______________

Stage I (Minimal) - 1-5
Stage II (Mild) - 6-15
Stage III (Moderate) - 16-40
Stage IV (Severe) - >40
Total____________

Laparoscopy________ Laparotomy________ Photography__________
Recommended Treatment__________________________
____
Prognosis____________________

PERITONEUM	**ENDOMETRIOSIS**	<1cm	1-3cm	>3cm
	Superficial	1	2	4
	Deep	2	4	6
OVARY	R Superficial	1	2	4
	Deep	4	16	20
	L Superficial	1	2	4
	Deep	4	16	20
	**POSTERIOR CULDESAC OBLITERATION**	Partial		Complete
		4		40
OVARY	**ADHESIONS**	<1/3 Enclosure	1/3-2/3 Enclosure	>2/3 Enclosure
	R Filmy	1	2	4
	Dense	4	8	16
	L Filmy	1	2	4
	Dense	4	8	16
TUBE	R Filmy	1	2	4
	Dense	4*	8*	16
	L Filmy	1	2	4
	Dense	4*	8*	16

***If the fimbriated end of the fallopian tube is completely enclosed, change the point assignment to 16.**

Denote appearance of superficial implant types as red [(R), red, red-pink, flamelike, vesicular blobs, clear vesicles], white [(W), opacifications, peritoneal defects, yellow-brown], or black [(B) black, hemosiderin deposits, blue]. Denote percent of total described as R___%, W___% and B___%. Total should equal 100%.

Additional Endometriosis: ______________________

Associated Pathology: ______________________

To Be Used with Normal Tubes and Ovaries

L R

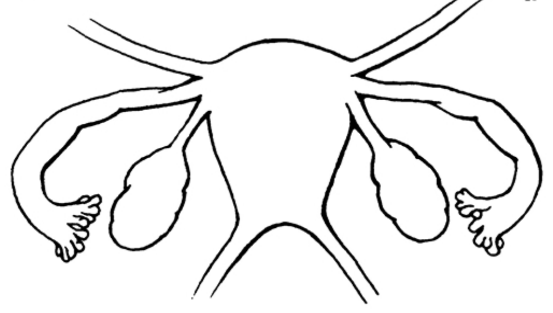

To Be Used with Abnormal Tubes and/or Ovaries

L R

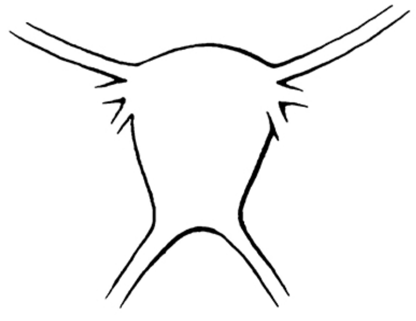

Figure S1: Revised American Society for Reproductive Medicine (rASRM) classification of endometriosis. Reprinted from the Revised American Society for Reproductive Medicine classification of endometriosis: 1996. Fertil Steril. 1997;67:817–21. *Copyright © 1997 American Society for Reproductive Medicine, with permission from Elsevier. All rights reserved.*
