# Supplementary material for: Non-invasive imaging techniques for diagnosis of pelvic deep endometriosis and endometriosis classification systems: an International Consensus Statement
Source: Facts Views Vis Obgyn. 2024 Jun 28;16(2):127–44. doi: 10.52054/FVVO.16.2.012 (PMC11366111; doi:10.52054/FVVO.16.2.012)
Supplement: Figure S2 — #Enzian classification system for women with superficial, ovarian and deep endometriosis. Reprinted from Keckstein et al. (2021), with permission from J. Keckstein. Copyright© 2021 The Authors. Published by John Wiley & Sons Ltd on behalf of Nordic Federation of Societies of Obstetrics and Gynecology (NFOG). Sacrouterine ligg/USL, uterosacral ligaments. [file FVVinObGyn-16-127-gs002.pdf]

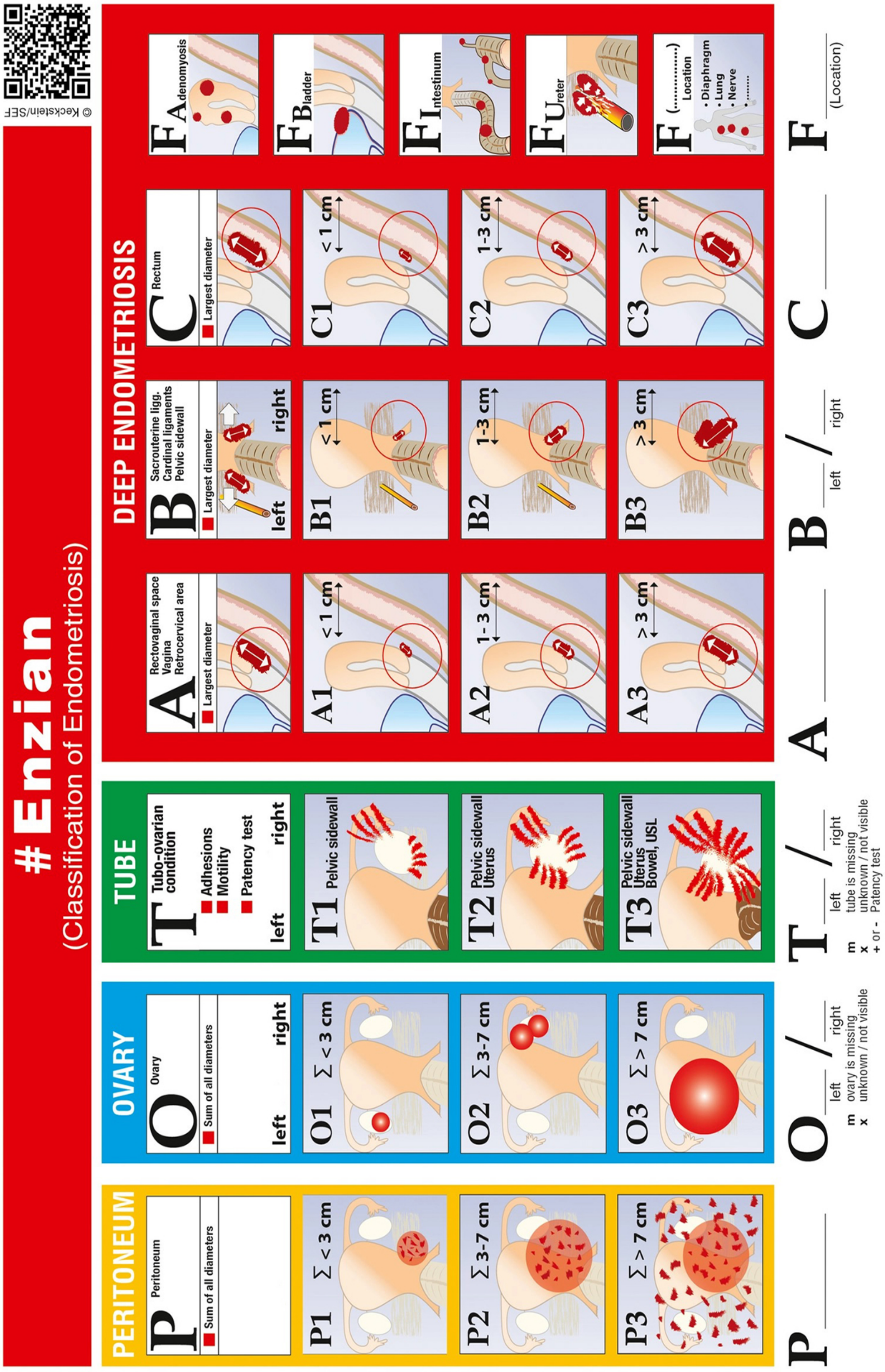

Figure S2: #Enzian classification system for women with superficial, ovarian and deep endometriosis. Reprinted from Keckstein et al. (2021), with permission from J. Keckstein. Copyright© 2021 The Authors. Published by John Wiley & Sons Ltd on behalf of Nordic Federation of Societies of Obstetrics and Gynecology (NFOG). Sacrouterine ligg/USL, uterosacral ligaments.
