# Supplementary material for: Non-invasive imaging techniques for diagnosis of pelvic deep endometriosis and endometriosis classification systems: an International Consensus Statement
Source: Facts Views Vis Obgyn. 2024 Jun 28;16(2):127–44. doi: 10.52054/FVVO.16.2.012 (PMC11366111; doi:10.52054/FVVO.16.2.012)
Supplement: Figure S3 — Ultrasound-based Endometriosis Staging System (UBESS), with sonographic features demonstrable on transvaginal ultrasound (TVS) and its prediction of level of surgical complexity. Adapted from Menakaya et al. (2016), with permission from ISUOG. SVG, sonovaginography. [file FVVinObGyn-16-127-gs003.pdf]

UBESS stage	Features demonstrable on TVS + gel SVG	Level of surgical complexity
Stage I	Normal mobile ovaries, absent non-bowel and absent bowel DIE, normal POD +/– SST	Level 1: negative laparoscopy or mild stage disease
Stage II	Endometrioma +/– immobile ovaries +/– non-bowel DIE +/– normal POD	Level 2: moderate stage disease
Stage III	Bowel DIE +/– immobile ovaries (endometriomas) +/– non-bowel DIE +/– normal POD	Level 3: higher stage disease

+/–,with or without;

DIE, deep infiltrating endometriosis; POD, pouch of Douglas; SST, site-specific tenderness.

Figure S3: Ultrasound-based Endometriosis Staging System (UBESS), with sonographic features demonstrable on transvaginal ultrasound (TVS) and its prediction of level of surgical complexity. Adapted from Menakaya et al. (2016), with permission from ISUOG. SVG, sonovaginography.
