# Supplementary material for: Non-invasive imaging techniques for diagnosis of pelvic deep endometriosis and endometriosis classification systems: an International Consensus Statement
Source: Facts Views Vis Obgyn. 2024 Jun 28;16(2):127–44. doi: 10.52054/FVVO.16.2.012 (PMC11366111; doi:10.52054/FVVO.16.2.012)
Supplement: Figure S4 — Endometriosis fertility index (EFI) system. This score predicts fertility outcome for women who attempt non-in-vitro fertilization conception following surgically documented endometriosis. Reprinted from Adamson and Pasta, 2010. Copyright© 2010 American Society for Reproductive Medicine, with permission from Elsevier. All rights reserved. AFS, American Fertility Society. [file FVVinObGyn-16-127-gs004.pdf]

ENDOMETRIOSIS FERTILITY INDEX (EFI) SURGERY FORM

LEAST FUNCTION (LF) SCORE AT CONCLUSION OF SURGERY

Score		Description		Left	Right
4	=	Normal	Fallopian Tube		
3	=	Mild Dysfunction			
2	=	Moderate Dysfunction	Fimbria		
1	=	Severe Dysfunction			
0	=	Absent or Nonfunctional	Ovary		

To calculate the LF score, add together the lowest score for the left side and the lowest score for the right side. If an ovary is absent on one side, the LF score is obtained by doubling the lowest score on the side with the ovary.

Lowest Score: ____ (Left) + ____ (Right) = ____ (LF Score)

ENDOMETRIOSIS FERTILITY INDEX (EFI)

Historical Factors			Surgical Factors		
Factor	Description	Points	Factor	Description	Points
Age			LF Score		
	If age is ≤ 35 years	2		If LF Score = 7 to 8 (high score)	3
	If age is 36 to 39 years	1		If LF Score = 4 to 6 (moderate score)	2
	If age is ≥ 40 years	0		If LF Score = 1 to 3 (low score)	0
Years Infertile			AFS Endometriosis Score		
	If years infertile is ≤ 3	2		If AFS Endometriosis Lesion Score is < 16	1
	If years infertile is > 3	0		If AFS Endometriosis Lesion Score is ≥ 16	0
Prior Pregnancy			AFS Total Score		
	If there is a history of a prior pregnancy	1		If AFS total score is < 71	1
	If there is no history of prior pregnancy	0		If AFS total score is ≥ 71	0
Total Historical Factors			Total Surgical Factors		

EFI = TOTAL HISTORICAL FACTORS + TOTAL SURGICAL FACTORS: ____ (Historical) + ____ (Surgical) = ____ (EFI Score)

ESTIMATED PERCENT PREGNANT BY EFI SCORE

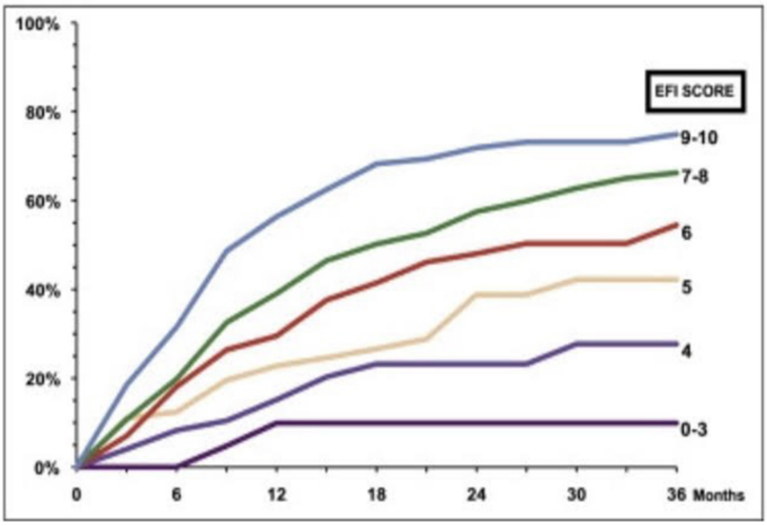

Figure S4: Endometriosis fertility index (EFI) system. This score predicts fertility outcome for women who attempt non-in-vitro fertilization conception following surgically documented endometriosis. Reprinted from Adamson and Pasta, 2010. *Copyright © 2010 American Society for Reproductive Medicine, with permission from Elsevier. All rights reserved.* *AFS, American Fertility Society.*
