# Supplementary material for: Non-invasive imaging techniques for diagnosis of pelvic deep endometriosis and endometriosis classification systems: an International Consensus Statement
Source: Facts Views Vis Obgyn. 2024 Jun 28;16(2):127–44. doi: 10.52054/FVVO.16.2.012 (PMC11366111; doi:10.52054/FVVO.16.2.012)
Supplement: Figure S5 — Magnetic resonance imaging (MRI) lexicon and deep pelvic endometriosis index (dPEI) classification: low extension (score 1 or 2), moderate extension (score 3 or 4) or severe extension (score 5 or more). Reproduced from Rousset et al. (2023). Copyright© 2022 The Author(s). Published by Elsevier Masson SAS on behalf of Société française de radiologie. All rights reserved. [file FVVinObGyn-16-127-gs005.pdf]

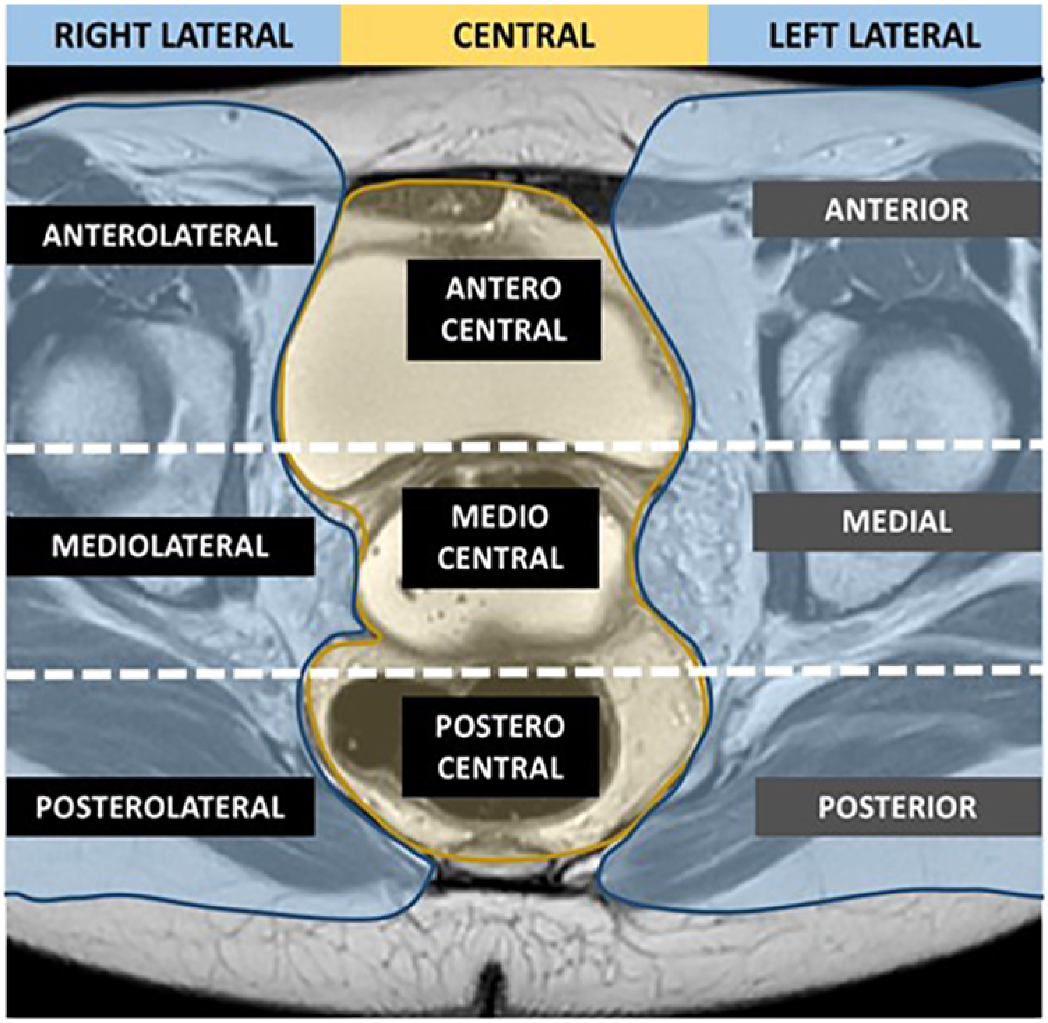

**Anterolateral**<br>Distal round ligament	**Anterocentral**<br>Proximal round ligament<br>Bladder	**Anterolateral**<br>Distal round ligament
**Mediolateral**<br>Parametrium<br>Ureter<br>Uterine artery<br>Visceral fascia and pelvic wall	**Mediocentral**<br>Torus and proximal USL<br>Posterior vaginal fornix<br>Rectovaginal septum<br>External myometrium adenomyosis	**Mediolateral**<br>Parametrium<br>Ureter<br>Uterine artery<br>Visceral fascia and pelvic wall
**Posterolateral**<br>Distal USL<br>Sacro-recto- genital septum	**Posterocentral**<br>Rectum and rectosigmoid junction	**Posterolateral**<br>Distal USL<br>Sacro-recto- genital septum

Extrapelvic
Caecum- ileum- appendix
Sigmoid colon
Abdominal wall
Inguinal regions
Ureters at the level of common iliac artery

Figure S5: Magnetic resonance imaging (MRI) lexicon and deep pelvic endometriosis index (dPEI) classification: low extension (score 1 or 2), moderate extension (score 3 or 4) or severe extension (score 5 or more). Reproduced from Rousset et al. (2023). *Copyright© 2022 The Author(s). Published by Elsevier Masson SAS on behalf of Société française de radiologie. All rights reserved.*
