# Supplementary material for: Non-invasive imaging techniques for diagnosis of pelvic deep endometriosis and endometriosis classification systems: an International Consensus Statement
Source: Facts Views Vis Obgyn. 2024 Jun 28;16(2):127–44. doi: 10.52054/FVVO.16.2.012 (PMC11366111; doi:10.52054/FVVO.16.2.012)
Supplement: Supplementary file 6 [file FVVinObGyn-16-127-a001.pdf]

Appendix I: Search strategy used for identification of potentially relevant studies with corresponding number of studies found (literature search using MEDLINE).

1	deep.mp.	281 819
2	endometriosis.mp. or exp Endometriosis/	30 750
3	1 and 2	2004
4	imaging.mp.	2 264 021
5	ultrasound.mp.	284 805
6	sonography.mp.	34 198
7	magnetic resonance.mp.	816 546
8	shift imaging.mp.	1092
9	exp Magnetic Resonance Imaging/	503 906
10	proton spin.mp.	735
11	spin echo.mp.	15 398
12	MRI.mp.	287 756
13	NMR.mp.	191 443
14	exp Tomography, X-Ray Computed/ or computed tomography.mp.	612 390
15	computer assisted tomography.mp.	824
16	beam tomography.mp.	566
17	Computerized Axial Tomography.mp.	1339
18	CT.mp.	392 841
19	CAT.mp.	123 972
20	4 or 5 or 6 or 7 or 8 or 9 or 10 or 11 or 12 or 13 or 14 or 15 or 16 or 17 or 18	3 080 240
21	3 and 20	692
