# Supplementary material for: Non-invasive imaging techniques for diagnosis of pelvic deep endometriosis and endometriosis classification systems: an International Consensus Statement
Source: Facts Views Vis Obgyn. 2024 Jun 28;16(2):127–44. doi: 10.52054/FVVO.16.2.012 (PMC11366111; doi:10.52054/FVVO.16.2.012)
Supplement: Supplementary file 7 [file FVVinObGyn-16-127-a002.pdf]

Appendix II: Levels of evidence and grades of statement used in this work(Guyatt et al., 2008; Howick et al., 2011).

Oxford Centre for Evidence-Based Medicine (CEBM) Levels of Evidence

1a: Systematic review (with homogeneity) of Level-1 diagnostic studies; or clinical decision rule with Level-1b studies from different clinical centres

1b: Validating cohort study with good reference standards; or clinical decision rule tested within one clinical centre

1c: Absolute SpPins and SnNouts*

2a: Systematic review (with homogeneity) of Level > 2 diagnostic studies

2b: Exploratory cohort study with good reference standards; or clinical decision rule after derivation or validated only on split-sample or databases.

3a: Systematic review (with homogeneity) of studies Level ≥ 3b

3b: Non-consecutive study; or without consistently applied reference standards.

4: Case–control study; poor or non-independent reference standard

5: Expert opinion without explicit critical appraisal, or based on physiology, bench research or 'first principles'

Grades of Statement

A (High): Further research is very unlikely to change our confidence in the estimate of effect.

- Several high-quality studies with consistent results
- In special cases: one large, high-quality multicentre trial

B (Moderate): Further research is likely to have an important impact on our confidence in the estimate of effect and may change the estimate.

- One high-quality study
- Several studies with some limitations

C (Low): Further research is very likely to have an important impact on our confidence in the estimate of effect and is likely to change the estimate.

- One or more studies with severe limitations

D (Very low): Any estimate of effect is very uncertain.

- Expert opinion
- No direct research evidence
- One or more studies with very severe limitations

Note: A minus sign '–' may be added to the level of evidence to denote evidence that fails to provide a conclusive answer because it is either: (a) a single result with a wide confidence interval; or (b) a systematic review with considerable heterogeneity. Such evidence is inconclusive, and therefore can only generate Grade-D recommendations. *'Absolute SpPin' is a diagnostic finding whose specificity is so high that a positive result rules in the diagnosis; 'Absolute SnNout' is a diagnostic finding whose sensitivity is so high that a negative result rules out the diagnosis.
